# Life Will Never Be the Same

**DOI:** 10.1002/1097-0061(20000930)17:3<241::AID-YEA28>3.0.CO;2-T

**Published:** 2000

**Authors:** M. A. Strivens

**Affiliations:** MRC Mammalian Genetics Unit and UK Mouse Genome Centre Harwell,Oxfordshire OX11 0RD UK


**Yeast**
*Yeast* 2000; **17**: 241–243.

**Meeting Review**

### Life will never be the same

### Annual Genome Sequencing and Biology Meeting, Cold Spring Harbor Laboratory, USA. May 2000

M.A. Strivens*
*MRC Mammalian Genetics Unit and UK Mouse Genome Centre, Harwell, UK*

*Correspondence to:
M. A. Strivens, MRC Mammalian Genetics Unit and UK Mouse Genome Centre, Harwell, Oxfordshire OX11 0RD,UK.
E-mail: mark@har.mrc.ac.uk*

It seems appropriate that the Cold Spring Harbor Genome Sequencing and Biology Meeting, which witnessed the creation of the Human Genome Organization (HUGO) in 1988, should this year present three major advances in genomic science: the completion of the finished sequence of *Drosophila melanogaster*; the announcement that 85% of the genome of *Homo sapiens* is now in draft sequence; and the complete, finished sequence of a second human chromosome, chromosome 21. Other major sessions of the meeting focused on single nucleotide polymorphisms (SNPs), ethical, legal and social implications (ELSI), as well as comparative and functional genomics.

There is significant debate as to whether the technique of 'whole genome shotgun' sequencing[1] is applicable to the elucidation of larger genomes (e.g. human). Prior to the beginning of this year, this technique had only been demonstrated on small microbial genomes (such as *Haemophilus influenzae*, with a genome of approximately 1.8 Mb [1]) and there had been considerable scepticism as to whether the technique would work in human or fruit fly. There was, therefore, considerable interest and excitement in the presentation by the Berkeley *Drosophila* Genome Project (BDGP) and Celera Genomics (Rockville, USA) on the full *Drosophila* sequence.

The crux of the whole-genome shotgun strategy is the assembly technique. Gene Myers (Celera Genomics) reported on how the 'double-barrelled' shotgun[2] approach had given a significant advantage to the computer algorithms employed in the assembly of the fly genome. The assembly system employs a bottom-up, nucleating strategy, initially assembling small islands of sequence of high confidence (diverting the assembly of repeat regions to later stages) and then searching for other sequence (including orientation data from clone end-sequencing) to join the islands together. In addition, he confirmed a less than 0.5% error rate in the automated assembly of repeat sequences that are a potential problem for this type of system.

Gerry Rubin (BDGP) and Mark Adams (Celera Genomics) presented material from the analysis of the genomic sequence, showing that the total number of genes is approximately 13 600 (compared with approximately 15 000 seen in the smaller 100 Mb *Caenorhabditis elegans* genome). In addition, there was substantial variation of 0–30 genes per 50 kb (but without the clustering seen in *C. elegans*). There was also a general trend showing a marked decrease in gene density, G + C content and an increase in transposons in the 1 Mb portion adjacent to the centromeric heterochromatin. Gerry

[1] Where all genomic material is sequenced as small fragments and resulting sequence fragments is reassembled by sophisticated software algorithms.

[2] This is where sequence is generated from both ends of shotgun clones as well as a range of small insert libraries. These libraries have a narrowly defined size range, so end-sequence data, provides important positional information for ordering and orientation of sequence fragments.

Copyright © 2000 John Wiley & Sons, Ltd.

Rubin commented that it was remarkable that the fly had only just over twice the number of genes compared with yeast, leading to the proposition that the complexity of an organism's gene content is not directly proportional to the complexity of the organism itself.

This observation prompted spirited debate, centred on exactly how many genes would comprise the human genome and, indeed, how one would define what comprised a single gene. In response to this debate, Ewan Birney of the European Bioinformatics Institute (EBI, UK) has created a sweepstake 'Gene Sweep' (**http://www.ensembl.org/genesweep.html**), allowing bets to be placed on what the eventual figure will be. It is characteristic of the current divergence in opinion that guesses currently range from 27 000 to 200 000. The sweepstake is open for another 2 years with definitions and absolute gene number being decided at the Cold Spring Harbor meetings in 2002 and 2003, respectively.

In addition to the announcement of the completion *Drosophila* genome, substantial progress has been achieved in the production of the working draft of the human genome. Jane Rogers (Sanger Centre, UK) presented the accelerated progress of the draft sequencing, now representing 85% of the human genome, with anything up to 97% in the final phase of checking (**http://www.sanger.ac.uk/HGP/stats.shtml**).

André Rosenthal (Institut für Molekulare Biotechnologie, Germany) reported the work of an international consortium of labs [2] producing a finished sequence for human chromosome 21. This chromosome has almost 20 disease loci associated with it, in addition to the trisomy that gives rise to Down's syndrome. From the analysis of the finished sequence, the consortium identified 127 genes of known function and 98 putative genes (including 59 pseudogenes). Approximately 40% of this chromosome is composed of repetitive elements, with some apparently very gene-poor areas totalling approximately 10 Mb. One of the most startling of the gene-poor regions was a 7 Mb region on 21q, where only one gene, as yet, has been detected. Using the number of genes identified from the completed sequence from human chromosomes 21 and 22, an extrapolation was done showing that (theoretically) the human genome could contain as little as 40 000 genes.

In addition to the intense experimental effort, several sessions focused on the bioinformatics techniques being designed to analyse the explosion of raw data from the sequencing and mapping centres. One of the principal problems with the high output of the various genome projects concerns the degree and appropriateness of annotation assigned to a sequence. This is exacerbated by the constantly evolving draft sequence. Ewan Birney (EBI, UK) presented the Ensembl (**http://www.ensembl.org/**) system, which is capable of defining features on draft sequence (assigning them stable identifiers), maintaining those features and identifiers as draft sequence progresses to its final finished form. This system is clearly a valuable tool in the early identification and exploitation of novel genes and regulatory elements. Comparative sequence analysis[3] is also becoming a viable tool for the dissection of novel genomic regions where sequence is available in a number of species. Greg Elgar (Human Genome Project Resource Centre, UK) presented comparative analysis between the compressed genome of the puffer-fish, *Fugu*, with a number of loci in mouse and man, demonstrating the power of this technique in identifying new genes and conserved regions in these species. In addition, to aid this type of approach, tools are being developed to align and visualize multi-species sequence comparisons, e.g. the Vista tool (Kelly Frazer, Lawrence Berkley National Laboratory, USA). This tool is capable of visualizing long-range alignments between several species and can be used to define statistical cut-offs for conserved elements.

The keynote speech was, appropriately, delivered by Francis Collins (National Human Genome Research Institute, USA), who has played a pivotal role in the organisation of the Human Genome Project and who clearly relished the prospect of delivering this address. He urged the assembled audience not to lose sight of the ultimate goals of the genome project; in the near-term this means the generation of high-quality, annotated sequence, that is made available to all. In the longer term, the requirement is to begin to use the data generated to pump-prime the next phase of genomic science – seeing that this, in turn, is translated into advances in basic medical science.

Given the potential impact of even the draft sequence on the whole of science and society, Dr

[3]The alignment of sequence from syntenic regions in order to identify evolutionary conserved regions, such as conserved exons and regulatory regions.

Copyright © 2000 John Wiley & Sons, Ltd. 

Collins, finished his address with 10 'exhortations' (see below) to the assembled scientists to 'attend to the broader social context' of the Human Genome Project. This involves both the education of school and college students and ensuring the understanding of the general public:

1. Cut out the lights, close the door, and get out of the lab. Spread your wisdom. Turn your bar napkins into genetic primers.
2. Volunteer to be a resource to local science teachers. Make yourself available to speak to elementary and secondary school students. Get involved in setting state and local science education standards. Alert local high school biology teachers to the NHGRI curriculum supplement on human genetic variation and HGP educational video documentary and CD ROM to be released this Fall.
3. Be an ambassador for science – speak to Rotary, Chamber of Commerce, church group or local bar association. Volunteer to provide technical assistance to local science museums and programmes.
4. Reward and foster community outreach activities by those who work for you, e.g. students, trainees and faculty.
5. Personalize scientific application, e.g. watch your language - make the Book of Life understandable to your Aunt Betty and nephew Jimmy.
6. Establish links with the schools of business, law, public health and education at your institution.
7. Start a DNA Day on your campus, at your hospital, or within your community.
8. Engage your community in a discussion of genetics. Use mistakes and over-simplifications in the press to improve local genetic literacy through letters to the editor. Write op-eds.
9. Get to know the ELSI issues and offer your expertise to state and federal policy makers. Share your concerns about the importance of protecting the privacy and fair use of genetic information.
10. Share ideas. Let me – and others – know what works in your community.

### Acknowledgements

My thanks to Francis Collins (NHGRI, USA) for providing me with extracts from his keynote lecture.

### References

1. Fleischmann RD *et al.* 1995. Whole-genome random sequencing and assembly of *Haemophilus influenzae*. *Science* **269**: 496–512.
2. Hattori M *et al.* 2000. The DNA sequence of human chromosome 21. The chromosome 21 mapping and sequencing consortium. *Nature* **405**: 311–319.

Copyright © 2000 John Wiley & Sons, Ltd.
